# Nutritional Evaluation of Housefly Larvae Meal in Broilers: Growth Performance, Gut Health, Metabolic Energy, and Microbiota Composition

**DOI:** 10.3390/ani16030386

**Published:** 2026-01-26

**Authors:** Kiyonori Kawasaki, Junliang Zhao, Bimala Sharma, Asia Khatun, Sharmin Sultana, Toshiya Kawasaki, Mitsuyoshi Ishikawa, Takuma Ban, Kiminobu Yano

**Affiliations:** 1 Faculty of Agriculture, Kagawa University, Miki 761-0795, Japan; 2 Department of Agriculture, Hetao College, Linhe 015000, China; 3 The United Graduate School of Agricultural Sciences, Ehime University, Matsuyama 790-0905, Japan; 4 Technology Development Division, Fujita Corporation, Atsugi 243-0125, Japan; 5 University Farm, Kagawa University, Sanuki 769-2304, Japan

**Keywords:** insects for feed, metabolizable energy, intestinal histology, short-chain fatty acids, cecal microbiota, alternative protein resources

## Abstract

Poultry production faces considerable challenges in terms of feed costs and sustainability, particularly regarding conventional protein sources such as fish meal (FM). Insect-based proteins, such as housefly larvae (HL) meal, offer sustainable alternatives. Therefore, this study aimed to investigate the effects of HL on growth, gut health, metabolizable energy, and gut microbiota of broilers to clarify the availability of HL meal for broiler diet. Experiment 1 was performed to investigate the effects of replacing FM with HL on growth performance, intestinal morphology, cecal short-chain fatty acids, and gut microbiota composition and diversity in male broilers. Experiment 2 was performed to assess the digestibility of broiler diets containing HL. HL addition effectively maintained growth and improved intestinal morphology. Blood analysis revealed that HL addition reduced TNF-α levels and improved protein utilization. Additionally, HL substitution induced significant changes in the gut microbiota community and metabolic pathways. In conclusion, HL are a viable alternative to FM with adequate energy content and beneficial effects on gut health, supporting their use as a sustainable protein source.

## Simple Summary

Fish meal (FM) is the most expensive ingredient in livestock feed owing to its relative scarcity and nutritional value. Recent research has shown that insects possess potential as sustainable and cost-efficient alternatives to FM in livestock nutrition. However, extensive research is necessary to identify suitable insect species; their optimal inclusion levels; and their effects on the growth performance, feed digestibility, and overall health of poultry species. In this study, we examined the effects of replacing FM with housefly larvae (HL) at levels of up to 5% on the growth performance, gut health, and overall health of broiler chickens. HL addition effectively maintained growth, improved intestinal morphology, reduced inflammation, enhanced protein utilization, and induced considerable changes in the gut microbiota community. Overall, this study highlights the potential of HL as a suitable replacement for FM in broiler diet.

## 1. Introduction

The global poultry industry faces unprecedented challenges in meeting the growing demand for animal protein and maintaining economic viability and environmental sustainability. The Food and Agriculture Organization (FAO) projects that global poultry production must increase by more than two-fold by 2050 to meet the protein needs of an estimated 10 billion people [1]. This expansion occurs against a backdrop of increasing feed costs, limited arable land for feed crop production, supply chain disruptions, and growing environmental concerns associated with conventional livestock production systems [2].

Traditional protein sources, particularly fish meal (FM), are considered premium ingredients in poultry feed formulations owing to their excellent amino acid profiles, high digestibility, and palatability [3]. FM is particularly valuable because of its high methionine content, an essential amino acid that is often limited to plant-based protein sources. However, the sustainability and cost-effectiveness of FM have become increasingly questionable owing to overfishing concerns, fluctuating availability, and volatile market prices, which can significantly affect feed costs [2]. Notably, these challenges have intensified the search for alternative protein sources that provide nutritional quality comparable to FM while offering improved sustainability and economic stability.

However, the development of alternative protein sources requires comprehensive nutritional evaluation, including precise determination of metabolizable energy (ME) values. ME represents the energy available to the animal for maintenance and production after accounting for losses in feces and urine, and is a critical parameter for feed formulation and cost analysis [4,5]. Accurate ME values are essential for optimizing feed formulations, predicting animal performance, and conducting economic evaluations of alternative ingredients in animal feed. Importantly, the practical application of novel feed ingredients remains limited without reliable ME data, regardless of their protein quality or nutritional attributes.

Among the various alternative protein sources, insect-based proteins have gained significant attention owing to their high protein content, favorable amino acid profiles, and potential for sustainable production of organic waste substrates [1,2]. Insects can efficiently convert low-value organic materials into high-quality protein biomass with minimal environmental impact [6]. Several insect species, including the black soldier fly (*Hermetia illucens*), yellow mealworm (*Tenebrio molitor*), and housefly (*Musca domestica*), have been evaluated as livestock feed, each possessing distinct nutritional profiles and production characteristics.

Housefly larvae (HL) have particular advantages in commercial applications that distinguish them from other insect species [7]. Compared with black soldier fly, which requires 14–21 days for larval development, HL have exceptionally rapid life cycles (a complete life cycle of 7–10 days under optimal conditions), enabling higher production turnover and more efficient biomass generation [7,8]. Additionally, HL demonstrate remarkable adaptability and can grow on various organic waste materials, including manure, food waste, and agricultural byproducts, with relatively simple rearing requirements and high feed conversion efficiency [2]. Moreover, the protein content of HL typically ranges from 50% to 65% on a dry matter basis, with a balanced amino acid profile suitable for monogastric animals. Notably, the fatty acid profile of housefly larvae exhibits a higher concentration of linoleic acid, an essential fatty acid for poultry, than that of other insect species [9]. Collectively, these characteristics make them more suitable as a feed component for chickens than black soldier fly, which predominantly contains lauric acid [10].

Despite these promising characteristics and the growing body of research on meals from other insects, such as black soldier flies and mealworms, there remains a significant research gap in the nutritional profile of HL meal. Although previous studies have focused primarily on black soldier fly larvae owing to regulatory approval in many regions [1], HL offer distinct advantages in terms of production efficiency and nutrient composition. Specifically, there is limited information on precise ME determination using standardized methodologies, systematic assessment of the effects of HL on intestinal morphology and gut health parameters, and comprehensive characterization of microbiota modulation and associated metabolic pathway changes in broilers fed with HL-supplemented diets. Furthermore, the integration of energy metabolism, gut health, and microbiome analyses in a single comprehensive study has not been previously conducted for HL, representing a critical knowledge gap hindering their practical application in commercial poultry production.

Recent findings on the gut health–performance axis have highlighted the importance of considering not only traditional nutritional parameters but also the effects of dietary interventions on intestinal morphology, microbiota composition, and microbial metabolic activities [11]. The gut microbiota plays a crucial role in nutrient digestion, immune function, and overall host health, and dietary modifications can induce substantial shifts in microbial community structure and metabolic capacity [12]. Short-chain fatty acids (SCFAs) produced by the gut microbiota through fermentation serve as important indicators of gut health and reflect the efficiency of nutrient utilization and protein fermentation in the digestive tract [13,14]. In particular, branched-chain fatty acids (BCFAs) are indicators of protein fermentation and can reveal the extent to which undigested proteins reach the hindgut, providing insights into protein digestibility and utilization efficiency [15].

Therefore, this study was designed as a comprehensive two-part investigation to address the research gaps in the evaluation of HL, under the hypothesis that HL could serve as a functional and energy-efficient alternative to FM in broiler diets. In particular, the first experiment examined the effects of partial and complete FM replacement with HL on growth performance, blood parameters, intestinal morphology, cecal SCFA profiles, and microbiota composition using advanced molecular techniques, including 16S rRNA gene sequencing and predictive functional profiling. Additionally, the second experiment focused on the precise determination of the ME value of HL meal using a standardized chromic oxide indicator methodology. Collectively, these experiments provide a complete nutritional profile essential for the practical application of HL meal in commercial broiler production, addressing both the nutritional adequacy and biological effects of this alternative protein source.

## 2. Materials and Methods

### 2.1. HL Preparation

HL were raised using a soybean-based food byproduct (okara) as the feed substrate. HL were collected at 4–6 days old, boiled in hot water at 100 °C for 3 min, and air-dried at 70 °C for 6 h.

### 2.2. Materials and Methods (Experiment 1)

#### 2.2.1. Animal Experiment Design and Diets (Experiment 1)

All animal experiments were approved by the Kagawa University Animal Experiment Committee (approval no.: KU-21677) and performed in accordance with the laws of the Japanese Association of Laboratory Animal Facilities of the National University Corporation. A total of 54 male Ross 308 broiler chicks (1-day-old) were obtained from a commercial hatchery (Mori Hatchery, Kan’onji Japan) and randomly allocated to nine pens (six birds per pen and three pens per treatment) using a completely randomized design. Birds were housed in battery cages measuring 1.8 m × 0.6 m × 0.75 m under standard environmental conditions (ambient temperature, between 12 and 26 °C; relative humidity, 50–70%) and had ad libitum access to food and water during the 50-day experimental period. Infrared ceramic heaters were installed, one in each pen, to maintain a brooding area temperature of approximately 40 °C for the young chicks.

In this experiment, the birds were randomly assigned to three experimental diets: control (C; 5% FM), low HL (L; 2.5% HL replacing 2.5% FM), and high HL (H; 5% HL completely replacing FM). All diets were formulated to be isocaloric and isonitrogenous according to broiler growth stages (Table 1). The nutrient requirements were met following the recommendations of the Japanese Feeding Standard for Poultry. The diets were then pelleted to 6 mm diameter using a pelletizer (HG-ZLSP150B, HAIGE, Ōra, Japan).

#### 2.2.2. Sample Collection (Experiment 1)

At 50 days of age, blood was collected from the brachial vein, and the birds were euthanized via cervical dislocation. Blood samples, organs, intestinal segments (duodenum, jejunum, and ileum), and cecal contents were collected.

#### 2.2.3. Blood Analysis

Hematological and plasma parameters were analyzed to examine the overall health of birds. Plasma biochemical parameters, including blood urea nitrogen, creatinine phosphokinase, glutamic oxaloacetic transaminase (GOT), lactate dehydrogenase (LDH), total cholesterol, and total protein, were assayed using an automatic biochemical analyzer (EZ SP-4430; ARKRAY, Kyoto, Japan). Additionally, plasma levels of tumor necrosis factor-alpha (TNF-α) were examined using a chicken TNF-α ELISA kit (Cloud-Clone Corp., Houston, TX, USA). All procedures were performed using specific kits according to the manufacturer’s instructions.

#### 2.2.4. Intestinal Morphological Analysis

Intestinal morphology was assessed as described by Kawasaki et al. [16]. Briefly, the small intestine was folded into eight sections to identify specific regions. Approximately 5 cm segments were excised from the duodenum (immediately distal to the stomach), the jejunum (at the third flexure), and the ileum (at the final flexure). Each segment was stretched and fixed in 10% neutralized formalin for 24 h. Subsequently, the fixed tissues were transferred to 70% ethanol and embedded in paraffin according to standard procedures. The paraffin-embedded tissues were sectioned at a thickness of 4 µm and subjected to hematoxylin and eosin staining. For morphometric analysis, villus height and crypt depth were measured at 10 randomly selected sites per animal using an optical microscope (BZ-9000; KEYENCE, Osaka, Japan) at ×10 magnification using ImageJ software (version 1.54f).

#### 2.2.5. 16S rRNA Gene Amplicon Sequencing and Taxonomic Analysis

DNA extraction, V3-V4 region of bacterial 16S rRNA gene amplicon sequencing, and taxonomic analysis were performed as described by Zhao et al. [17].

#### 2.2.6. Cecal SCFA Analysis

Briefly, the concentration of SCFAs in the cecal content was determined using a GCMS-QP2010 SE system (Shimadzu, Kyoto, Japan) equipped with an Agilent DB-5MS column, as described by Kawasaki et al. [18].

#### 2.2.7. Prediction of Microbial Functional Abundance and Metabolic Pathway Analysis

Prediction of metagenome function and pathway analysis were conducted using the Qiime2 (version 2024.5) [19] and PICRUSt2 algorithms (version 2024.5) [20], as described by Zhao et al. [17]. Briefly, the metagenomic functional profiles were annotated using the Kyoto Encyclopedia of Genes and Genomes (KEGG) database. Thereafter, the obtained KEGG orthologs were subjected to principal component analysis (PCA) and KEGG pathway annotation. The KEGG pathway enrichment ratio was calculated as the number of genes expressed in individuals relative to the total number of genes in each pathway.

#### 2.2.8. Statistical Analysis

Data were analyzed using appropriate statistical methods depending on the data distribution. The normality of the data was assessed using the Shapiro–Wilk test, and the homogeneity of variance was evaluated using Levene’s test. Normally distributed data were analyzed using one-way analysis of variance (ANOVA), followed by Tukey’s test for multiple comparisons. Non-normally distributed data were analyzed using the Kruskal–Wallis test with Bonferroni post hoc correction. For microbiota analysis, PERMANOVA was used to test differences in community composition. Statistical significance was set at *p* < 0.05, and a trend toward significance was considered at 0.05 ≤ *p* < 0.1. Data are presented as mean ± standard error (SE), unless otherwise specified. Statistical analyses and figures were generated using R version 4.3.1 (R Foundation for Statistical Computing, Vienna, Austria). Specifically, the ‘vegan’ package (version 2.7-2) was used for PERMANOVA analysis.

### 2.3. Materials and Methods (Experiment 2)

#### 2.3.1. Animal Experiment Design and Diets (Experiment 2)

The animal experiments were approved by the Kagawa University Animal Experiment Committee (approval no. KU-24668) and conducted according to the laws of the Japanese Association of Laboratory Animal Facilities of the National University Corporation. Twenty male Ross 308 broiler chicks (1-day-old) were purchased from a commercial hatchery (Mori Hatchery, Kan’onji, Japan) and reared on commercial diets until 4 weeks of age. During the experimental period, 20 birds were individually housed in metabolic cages designed for precise excreta collection (ambient temperature, between 18 and 22 °C; relative humidity, 50–70%). The birds were arranged such that two birds shared access to a single feeding box, resulting in an effective sample size of *n* = 5 per treatment group. Birds were allowed a 7-day adaptation period to acclimatize to the metabolic cages and experimental conditions before the start of data collection.

During the experiment, the birds were assigned to two treatment diets: a basal diet group and a test diet group containing 20% HL meal substituting for 20% of the basal diet (80:20 ratio). Both diets contained 0.1% chromium oxide (Cr_2_O_3_) as an indigestible marker for digestibility calculation. The basal diet was formulated according to the Japanese Feeding Standard for Poultry and consisted primarily of corn, soybean meal, and supplementary nutrients (Table 2).

#### 2.3.2. Sample Collection (Experiment 2)

The experimental diets were fed for 8 days, and fecal samples were collected during the final 3 days. Feed was provided in measured quantities to minimize waste, and fecal samples were collected twice daily (morning and evening) from each bird, with careful removal of feed contamination. Collected fecal samples were immediately weighed, combined by day for each bird, and dried at 60 °C for 2 days under forced air circulation. After drying, the 3-day collections were pooled, ground into a fine powder, and stored for chemical analysis.

#### 2.3.3. Proximate and Chemical Analysis

All feed ingredients, experimental diets, and excreta samples were analyzed according to the Feed Analysis Standards of the Ministry of Agriculture, Forestry, and Fisheries, Japan. Moisture and nitrogen contents were determined by oven-drying and the Kjeldahl method, respectively. Gross energy was determined using a bomb calorimeter. Additionally, chromium oxide concentrations in the fecal samples were determined using a colorimetric method with potassium phosphate reagent. All analyses were conducted at the Japan Scientific Feeds Association (https://kashikyo.lin.gr.jp/; accessed on 30 January 2025).

#### 2.3.4. ME Calculations

ME was determined using an indicator method with Cr_2_O_3_ as an indigestible marker, as described by Vogtmann et al. [21] and Sales and Janssens [22]. The ratio of excreta output to feed intake (*EO/FI*) was estimated from the concentration of chromic oxide in the feed and excreta according to the following equation:

$$\frac{EO}{FI}=\frac{{Cr}_{feed}}{{Cr}_{excreta}}$$

where *Cr_feed_* and *Cr_excreta_* represent the chromic oxide concentrations in the feed and excreta, respectively.

The apparent ME (AME) of each diet (kcal/kg) was calculated as follows:

$$AME={GE}_{feed}-\frac{{Cr}_{feed}}{{Cr}_{excreta}}\times {GE}_{excreta}$$

where *GE_feed_* and *GE_excreta_* denote the gross energy content of the feed and excreta, respectively, measured on the same basis (dry matter).

The nitrogen-corrected *AME* (AMEn) was computed by correcting for nitrogen retention, following the procedure described by Hill and Anderson [4].

$$AMEn=AME-8.22\times \left(N_{feed}-\frac{{Cr}_{feed}}{{Cr}_{excreta}}\times N_{excreta}\right)$$

where *N_feed_* and *N_excreta_* represent the nitrogen concentrations in the feed and excreta, respectively. The factor 8.22 kcal/g corresponds to the energy value associated with nitrogen retention in poultry.

The ME of the basal and test diets were first calculated, and then the ME of the HL meal was determined using the difference method as follows:

$$ME\;of\;housefly\;larvae\;(Mcal/kg)=\frac{{AMEn}_{test}-(1-p)\times {AMEn}_{basal}}{p}\;Metabolizability\;(\%)\;=\;[ME\;of\;housefly\;larvae/GE\;of\;housefly\;larvae]\;\times \;100$$

where *AMEn_test_*, *AMEn_basal_*, and *p* represent the ME of the test and basal diets and the concentration of HL in the test diet, respectively.

## 3. Results

### 3.1. Results (Experiment 1)

#### 3.1.1. Growth Performance and Feed Efficiency

Notably, substituting FM with HL meal did not significantly affect body weight gain, feed intake or feed conversion ratio (FCR) during the experimental period (*p* > 0.05; Table 3). Although not statistically significant, body weight was higher in the L group than in the C group at certain time points. In contrast, birds in the H group exhibited a slight reduction in body weight compared with those in the control group. Importantly, no adverse health effects were observed in any treatment group.

#### 3.1.2. Organ Indices

No significant differences were observed in the relative organ weights (liver, spleen, kidney, and intestine) among the treatment groups (*p* > 0.05; Table 4). However, intestinal length tended to decrease in the HL-fed group compared to the control group (*p* = 0.072; Table 4).

#### 3.1.3. Blood Biochemistry and Hematology

Total cholesterol levels were significantly higher in both HL groups than in the control group (*p* < 0.05; Table 5). Although GOT and LDH activities were significantly higher in the HL-fed groups, they remained within the normal physiological ranges. TNF-α levels were significantly downregulated in the H group (*p* < 0.05; Table 5). Hematological analysis revealed a significant increase in lymphocyte percentage and red blood cell indices (MCH and MCHC) in the HL-fed groups (*p* < 0.05; Table 6). However, granulocyte percentage was significantly lower in the HL-fed groups compared to the control group (*p* < 0.05; Table 6).

#### 3.1.4. Intestinal Morphology

Morphological analysis revealed consistent improvements in villus architecture across all intestinal segments in the HL-fed groups. In the duodenum, villus height was significantly higher in the H group (1773.49 ± 53.17 μm) than in the control group (1606.31 ± 50.01 μm, *p* < 0.05), with corresponding improvements in the villus height-to-crypt depth ratio (12.18 ± 0.48 vs. 10.39 ± 0.41, *p* < 0.05). Similar beneficial patterns were observed in the jejunum and ileum, with significant improvements in villus height-to-crypt depth ratios in the HL groups compared with the control group (*p* < 0.05; Table 7).

#### 3.1.5. Cecal Microbiota Composition

**Figure 1:**
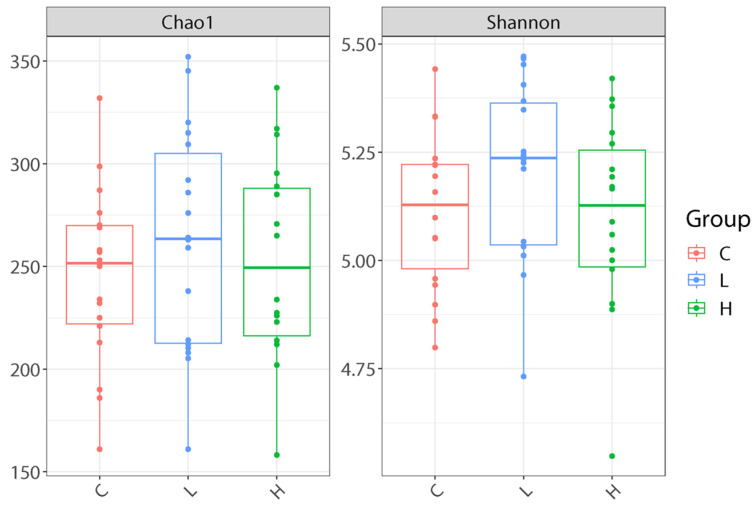
Alpha diversity of cecal microbiota. Alpha diversity indices (Shannon index and observed species) showed no significant differences among the treatment groups (Kruskal–Wallis pairwise *p* > 0.05), indicating that the overall bacterial richness and diversity were maintained across all dietary treatments. C diet: 3.0% fish meal and 0% housefly larvae (HL) meal; L diet: 1.5% fish meal and 1.5% HL meal; H diet: 0% fish meal and 3.0% HL meal.

**Figure 2:**
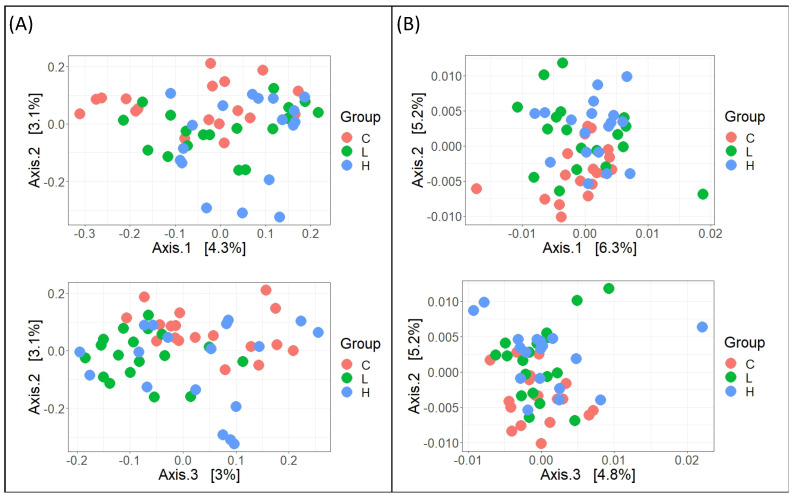
Beta diversity of cecal microbiota. Principal coordinate analysis (PCoA) based on unweighted UniFrac (**A**) and weighted UniFrac (**B**) distances revealed distinct clustering of samples by treatment group. PERMANOVA pairwise analysis confirmed significant differences in microbial community structure ((**A**): *p* = 0.001 and (**B**): *p* = 0.001). C diet: 3.0% fish meal and 0% housefly larvae (HL) meal; L diet: 1.5% fish meal and 1.5% HL meal; H diet: 0% fish meal and 3.0% HL meal.

Microbiota analysis revealed no significant differences in alpha diversity indices (Chao1 and Shannon) among the groups (Figure 1). However, beta diversity analysis demonstrated significant differences in microbial community structure. Both unweighted and weighted UniFrac distances revealed a clear separation between the treatment groups (PERMANOVA = 0.001 and 0.001, respectively; Figure 2), indicating that dietary HL inclusion restructured the cecal bacterial ecosystem and maintained overall species diversity.

#### 3.1.6. SCFA Concentrations

SCFA analysis revealed significant treatment-specific changes in cecal fermentation profiles. Although acetic, propionic, butyric, and valeric acids showed no significant differences among the groups (*p* > 0.05), isobutyric and isovaleric acids showed dose-dependent reductions with increasing HL inclusion levels (*p* < 0.05; Table 8).

#### 3.1.7. Predicted Metabolic Pathways

PICRUSt2 functional analysis identified 78 significantly different metabolic pathways among the treatment groups (*p* < 0.05; Table S1). Importantly, the most significantly altered pathways were those involved in carbohydrate and amino acid biosynthesis, vitamin production, and energy metabolism. Additionally, the low-HL group showed the most pronounced upregulation of beneficial metabolic pathways, particularly those involved in carbohydrate fermentation and vitamin synthesis (Table 9). 

### 3.2. Results (Experiment 2)

#### ME Determination

In this study, we investigated the energy value and digestibility of diets containing HL in broiler chickens. All birds remained healthy throughout the experimental period, and no adverse effects were observed during dietary treatment. Digestibility experiments revealed that HL meal had an ME of 4.43 ± 0.10 Mcal/kg, with a metabolizability of 72.0 ± 1.7% (Table 10). Individual bird responses ranged from 4.05 to 4.60 Mcal/kg, indicating some variability in digestibility among animals. However, the mean value indicated good energy availability in the diets for broilers.

## 4. Discussion

### 4.1. Growth Performance and Physiological Adaptations

In the present study, substitution with HL meal effectively maintained growth performance and feed efficiency, demonstrating the nutritional adequacy of this alternative protein source for broiler production. Our findings align with those of previous studies on insect-based proteins, which have generally shown performances comparable to traditional protein sources. For instance, Biasato et al. [23] reported that the partial replacement of soybean meal with yellow mealworm (*Tenebrio molitor*) larval meal did not compromise the growth performance of chickens. Similarly, Cullere et al. [24] found satisfactory growth performance in quails fed with black soldier fly (*Hermetia illucens*) meals. Overall, these results support the viability of insects as reliable protein sources for poultry feed.

Additionally, the slight elevations in serum cholesterol, GOT, and LDH levels in HL-fed birds (values remained within the normal physiological range) likely reflect metabolic adaptations to different lipid and protein profiles. Insect meals, including HL, often have distinct fatty acid profiles rich in saturated fatty acids, such as lauric acid, which can influence lipid metabolism and cholesterol synthesis. Józefiak et al. [25] observed changes in the lipid parameters of birds fed with insect-supplemented diets, which were attributed to the specific lipid composition of the larvae. Elevated GOT and LDH levels, although non-pathological, may indicate increased protein turnover and amino acid catabolism required for the efficient utilization of insect proteins, a phenomenon often associated with high-protein poultry diets.

Notably, the reduction in TNF-α levels in the high-HL group suggests potential anti-inflammatory benefits of HL. This effect may be mediated by the bioactive components of HL, such as antimicrobial peptides (AMPs) and chitin. Insect-derived peptides possess immunomodulatory properties that can downregulate proinflammatory cytokines [26]. This anti-inflammatory potential may contribute to improved gut health and performance stability by reducing the metabolic cost of immune system activation and allowing more energy to be directed towards growth [27].

### 4.2. Intestinal Health and Morphological Improvements

In the present study, the consistent improvements in intestinal morphology across all intestinal segments demonstrated the health benefits of HL in the gut. Enhanced villus architecture directly translates to an increased absorptive surface area and improved nutrient absorption capacity. Similarly, Marono et al. [28] reported that the inclusion of insect meal can positively influence gut morphology in poultry. Chitin, a fibrous polysaccharide found in the exoskeleton of insects, is believed to play a crucial role in this process [29]. Although indigestible by avian enzymes, low levels of chitin can act as a physical stimulus for the gut mucosa, promoting villus growth and cell proliferation [23]. Moreover, Ipema et al. [30] highlighted the role of insect components in modulating gut health, suggesting that these bioactive molecules create a more favorable intestinal environment for nutrient absorption.

### 4.3. Microbiota Composition and Functional Adaptations

In this study, the preservation of alpha diversity and significant changes in beta diversity indicate that HL meal promotes beneficial restructuring of the gut microbiota rather than disruptive changes. Dietary inclusion of insect meal introduces novel substrates such as chitin, which stimulates specific bacterial taxa capable of chitinolysis. Kawasaki et al. [16] identified specific chitin-degrading bacteria that proliferate in response to insect-based diets. This shift in community composition is comparable to the effects of prebiotic intervention.

Importantly, the prebiotic potential of chitin and its derivative, chitosan, is a key factor in these microbiota shifts. These compounds can be fermented by the cecal microbiota, promoting the growth of beneficial genera (*Lactobacillus* and *Bifidobacterium*) and inhibiting potential pathogens. Saviano et al. [31] observed similar positive shifts in the gut microbiome of dogs fed with insect-based diets, reinforcing the cross-species potential of insect meals as functional feed ingredients. Moreover, the 78 significantly altered metabolic pathways identified in our study demonstrated the enhanced functional capacity of this restructured microbiota, particularly in carbohydrate metabolism and vitamin production, which may compensate for the nutritional differences between HL and FM.

### 4.4. SCFA Profile and Protein Utilization Efficiency

The significant dose-dependent reduction in BCFAs (isobutyric and isovaleric acids) provides crucial insights into protein utilization efficiency. BCFAs are primarily produced by bacterial fermentation of branched-chain amino acids (valine, leucine, and isoleucine) derived from undigested proteins that reach the cecum [32]. A reduction in cecal BCFAs implies that fewer undigested proteins enter the hindgut, indicating improved protein digestibility and absorption in the small intestine. This is supported by the observed improvements in intestinal morphology, particularly the increased villus height, which facilitates amino acid uptake.

High protein digestibility is crucial for growth performance and environmental sustainability. Improved upper tract digestibility reduces nitrogen excretion and the environmental footprint of poultry production. Batista et al. [33] emphasized that enhanced protein digestibility in insect-fed birds leads to better nitrogen retention and reduced emission of ammonia. Similarly, Elahi et al. [34] reported that replacing fishmeal with insect meal improved the apparent ileal digestibility of crude protein and amino acids. Sedgh-Gooya et al. [35] further demonstrated that optimized protein digestion is directly linked to gut health and reduced pathogen proliferation in the hindgut, as excess protein in the cecum can fuel the growth of proteolytic pathogens, such as *Escherichia coli*.

### 4.5. ME Value and Nutritional Implications

In this study, the ME value of 4.43 ± 0.10 Mcal/kg positions HL meal favorably compared to conventional protein sources and other insect meals. This value is comparable to that of high-quality protein ingredients and exceeds the values reported for other insect species. For example, Schiavone et al. [36] reported AMEn values for black soldier fly larvae meal ranging from 3.38 to 3.96 Mcal/kg depending on the fat content. The higher energy value observed for HL in our study may be attributed to its specific fatty acid profile and high digestibility.

Several factors influence ME determination of insect meals, including the larval stage at harvest, substrate used for rearing, and processing methods (e.g., defatting). Finke [37] highlighted that the nutrient composition of insects is highly variable and dependent on these factors. Our results suggest that HL processing via boiling and air-drying preserves energy density and availability. A metabolizability of 72.0% indicates efficient energy partitioning and that a significant portion of the gross energy is available for maintenance and production. De Marco et al. [38] reported similarly high metabolizability values for various insect meals, reinforcing their potential as energy-dense feed ingredients. The individual bird variation in ME values (4.05–4.60 Mcal/kg) reflects normal biological variation and suggests that while most birds efficiently utilize HL meal, formulation strategies should account for this variability to ensure consistent flock performance.

### 4.6. Bioactive Compounds and Health Benefits

In addition to basic nutrients, HL meal contains bioactive compounds that confer health benefits. HL are a rich source of lauric acid, a medium-chain fatty acid with proven antimicrobial properties. Lauric acid disrupts the cell membranes of pathogenic bacteria, thereby reducing the gut bacterial load. Spranghers et al. [39] discussed the antimicrobial potential of insect fats and noted that high concentrations of lauric acid were particularly effective against Gram-positive bacteria.

Insects also possess a potent immune system that relies on AMPs such as defensins and cecropins. These peptides are retained in the diet and exert immunomodulatory effects on the host. Park et al. [40] demonstrated that insect-derived AMPs can enhance immune responses and disease resistance in livestock. The presence of these bioactive compounds likely contributes to the observed reduction in inflammatory markers (TNF-α) and improvement in gut health, suggesting that HL meal also acts as a functional ingredient.

### 4.7. Economic and Practical Implications

The determined ME value of 4.43 Mcal/kg, combined with the demonstrated protein utilization benefits, suggests that HL meal can be economically competitive with conventional protein sources when properly valued for its energy content. As feed costs constitute the majority of poultry production expenses, incorporating energy-dense alternatives such as HL into feed formulations can optimize profitability. Oonincx et al. [6] conducted a life cycle assessment and showed that insect production can be more sustainable and potentially cost-effective than traditional livestock feed ingredients, particularly when reared on organic waste streams.

However, their scalability and industrial production remain challenging. Although small-scale production is feasible, scaling up to meet the demands of the global poultry industry requires significant investment in automated rearing and processing technologies. Van Huis [41] emphasized that regulatory frameworks and consumer acceptance are critical factors for the widespread adoption of insects as feed. However, the ability of houseflies to convert low-value organic waste into high-value biomass presents a circular economic opportunity. Berggren et al. [42] argued that insect bioconversion systems offer a viable solution for sustainable waste management and feed production, potentially stabilizing feed prices in the long term.

### 4.8. Integration of Experimental Findings

The integration of findings from both experiments provides a comprehensive understanding of the nutritional value and biological effects of HL meals from a systems biology perspective. The adequate ME value (4.43 Mcal/kg) supports the maintained growth performance observed in the feeding trial, whereas the improved intestinal morphology explains the enhanced digestibility reflected in the 72.0% metabolizability. The correlation between reduced levels of BCFAs and upregulated amino acid biosynthesis pathways suggests a coordinated adaptation in which improved small intestinal protein digestion reduces cecal protein fermentation, whereas enhanced microbial amino acid production provides supplementary nutritional benefits.

This reflects the complex host–microbiota–diet triangle described by Jha and Berrocoso [11], in which dietary components modulate the gut microbiota, which influences host physiology and immune status. The restructured microbiota with enhanced metabolic capabilities appears to optimize energy and nutrient utilization from HL meal, potentially explaining how nutritional adequacy is maintained despite differences in amino acid and mineral profiles. This microbiota-mediated adaptation is a novel mechanism that supports the successful substitution of conventional protein sources with insect-based alternatives.

Although this study provides a comprehensive evaluation of HL, some limitations should be acknowledged. For instance, the disparity in mineral content between HL and FM, particularly calcium and phosphorus deficiencies, requires careful attention during feed formulation. Insect meals are generally low in calcium and have imbalanced Ca/P ratios. Meneguz et al. [43] emphasized the importance of balancing mineral intake when formulating insect-based diets to prevent skeletal issues. Future research should investigate optimal supplementation protocols and evaluate the long-term effects of HL meal inclusion on skeletal development and overall health throughout extended production cycles.

Furthermore, the presence of anti-nutritional factors (chitin) warrants further investigation, as excessive chitin can negatively affect digestibility. Regulatory considerations regarding the substrates used to rear insects must be addressed to ensure food safety. Finally, studies are needed to examine the impact of HL on meat quality, sensory attributes, and shelf life. Benzertiha et al. [44] reported that although insect meals generally do not negatively affect meat quality, the specific fatty acid profile of larvae can influence the fatty acid composition of broiler meat, which is a critical parameter for consumer acceptance.

## 5. Conclusions

Our study demonstrated that HL is a viable and beneficial alternative to FM in broiler diets. Additionally, the ME value of 4.43 ± 0.10 Mcal/kg with 72.0% metabolizability indicates good energy availability and digestibility, supporting the growth performance and feed efficiency observed in the feeding trial.

The integration of growth performance data, metabolic energy analysis, and advanced gut health assessments revealed multiple mechanisms supporting the nutritional adequacy of HL. Improved intestinal morphology may enhance nutrient absorption capacity. In contrast, restructured microbiota with enhanced metabolic capabilities provide supplementary nutritional benefits through increased amino acid biosynthesis and vitamin production pathways. Notably, pathway analysis revealed that the L group, characterized by a specific replacement ratio of HL for FM, exhibited the most favorable metabolic profiles, suggesting an optimal balance for nutrient utilization. Moreover, the significant reduction in BCFAs may demonstrate improved protein utilization efficiency, and the maintenance of beneficial SCFAs indicates the preservation of gut health. Collectively, these findings suggest that HL meal can replace the nutritional contributions of FM and provide gut health benefits that could improve overall production efficiency and bird welfare. Furthermore, the economic implications of this study are promising. In particular, the determined ME values support cost-effective feed formulations. However, careful attention must be paid to mineral supplementation, particularly of calcium and phosphorus, when replacing FM with HL. Future research should focus on optimizing mineral supplementation protocols and evaluating the long-term production performance to fully realize the potential of HL meal as a sustainable protein source for commercial broiler production.

## Figures and Tables

**Table 1:** Feed formulation and proximate nutrients on the diets in experiment 1.

Ingredients (% in Fresh Matter)	Grower Feed			Finisher Feed		
	C	L	H	C	L	H
Maize grain	55.5	55.1	54.8	59.3	58.9	58.5
Rice bran (defatted)	6.7	6.7	6.6	7.2	7.1	7.1
Corn gluten meal	3.8	3.8	3.8	4.1	4.1	4.1
Soybean meal	21.0	21.5	22.0	16.0	16.5	17.0
Fish meal	5.0	2.5	0.0	5.0	2.5	0.0
Housefly larvae meal	0.0	2.5	5.0	0.0	2.5	5.0
Dried distillers’ grains	3.7	3.7	3.7	4.0	3.9	3.9
Corn oil	2.2	2.2	2.2	2.4	2.4	2.3
Tricalcium phosphate	1.3	1.3	1.3	1.4	1.4	1.4
Premix ^1^	0.4	0.4	0.4	0.5	0.5	0.5
Salt	0.1	0.1	0.1	0.1	0.1	0.1
Calcium carbonate	0.1	0.1	0.1	0.1	0.1	0.1
Proximate nutrients (% in dry matter) and energy content						
Dry matter ^2^	88.10	88.31	87.90	87.46	87.58	87.80
Crude protein ^2^	19.11	18.56	19.80	17.14	16.94	17.76
Crude fat ^2^	3.23	3.06	3.02	3.43	3.42	3.58
Crude fiber ^2^	3.88	4.00	4.46	3.10	3.65	3.80
Crude ash ^2^	6.21	6.12	4.81	6.03	5.46	4.96
NFE ^3^	55.67	56.57	55.81	57.76	58.11	57.7
ME ^3,4^ (Mcal/kg)	3.37	3.37	3.39	3.40	3.41	3.44

NFE: nitrogen-free extract. ^1^ Premix: water (3.4%); Na (300.00 g/kg); Fe (8.20 g/kg); Zn (2.20 g/kg); Mn (10.00 g/kg); Cu (0.62 g/kg); choline (30.00 g/kg); vitamin A (3,000,000 IU/kg); vitamin B_1_ (0.14 g/kg); vitamin B_2_ (0.35 g/kg); vitamin B_3_ (1.40 g/kg); vitamin B_5_ (0.50 g/kg); vitamin B_6_ (0.25 g/kg); vitamin B_7_ (0.50 g/kg); vitamin B_9_ (0.025 g/kg); vitamin B_12_ (0.0005 g/kg); vitamin D (600,000 IU/kg); vitamin E (596 IU/kg). ^2^ Analyzed data. ^3^ Calculated data. ^4^ ME = (Crude protein × 3.84 + Crude fat × 9.33 + NFE × 4.2)/100.

**Table 2:** Composition of basal diet for metabolizable energy determination.

Ingredient	Inclusion Rate (%)
Corn (ground)	46.00
Grain sorghum	12.00
Soybean meal	30.53
Defatted rice bran	3.00
Corn gluten meal (60% CP)	5.00
Dicalcium phosphate	1.10
Calcium carbonate	1.05
Salt	0.30
L-Lysine HCl	0.22
DL-Methionine	0.35
Choline chloride	0.12
Premix ^1^	0.30
Selenium yeast	0.03

^1^ Premix: water (3.4%); Na (300.00 g/kg); Fe (8.20 g/kg); Zn (2.20 g/kg); Mn (10.00 g/kg); Cu (0.62 g/kg); choline (30.00 g/kg); vitamin A (3,000,000 IU/kg); vitamin B_1_ (0.14 g/kg); vitamin B_2_ (0.35 g/kg); vitamin B_3_ (1.40 g/kg); vitamin B_5_ (0.50 g/kg); vitamin B_6_ (0.25 g/kg); vitamin B_7_ (0.50 g/kg); vitamin B_9_ (0.025 g/kg); vitamin B_12_ (0.0005 g/kg); vitamin D (600,000 IU/kg); vitamin E (596 IU/kg).

**Table 3:** Body weight gain, feed intake, and FCR during the experiment.

Parameters	C	L	H	*p*-Value
Body weight gain during Grower period (1w–4w)	1189.8 ± 17.4	1141.7 ± 19.2	1019.0 ± 53.2	0.058
Body weight gain during Finisher period (4w–7w)	1422.3 ± 165.3	1748.9 ± 109.4	1488.7 ± 108.9	0.193
Body weight gain during experiment (1w–7w)	2612.1 ± 182.4	2890.5 ± 99.0	2507.6 ± 126.5	0.177
Feed intake during Grower period (1w–4w)	1808.6 ± 51.0	1813.7 ± 15.9	1641.1 ± 26.6	0.066
Feed intake during Finisher period (4w–7w)	3564.9 ± 372.2	4216.2 ± 289.7	3586.1 ± 306.2	0.193
Feed intake during experiment (1w–7w)	5373.5 ± 419.6	6029.9 ± 302.4	5227.2 ± 319.7	0.177
FCR during Grower period (1w–4w)	1.52 ± 0.02	1.59 ± 0.04	1.62 ± 0.06	0.301
FCR during Finisher period (4w–7w)	2.51 ± 0.04	2.41 ± 0.04	2.41 ± 0.03	0.177
FCR during experiment (1w–7w)	2.06 ± 0.02	2.08 ± 0.04	2.08 ± 0.04	0.837

C diet: 3.0% fish meal and 0% housefly larvae (HL) meal; L diet: 1.5% fish meal and 1.5% HL meal; H diet: 0% fish meal and 3.0% HL meal. Data are presented as mean ± standard error (SE; *n* = 3). Significant differences were set at *p* < 0.05, and trends were defined as 0.05 ≤ *p* < 0.10.

**Table 4:** Organ indices (% of body weight) and small intestinal length.

Parameter	C	L	H	*p*-Value
Liver (%)	1.82 ± 0.06	1.74 ± 0.03	1.70 ± 0.04	0.231
Spleen (%)	0.11 ± 0.01	0.13 ± 0.01	0.11 ± 0.01	0.283
Kidney (%)	0.38 ± 0.03	0.43 ± 0.02	0.42 ± 0.02	0.268
Intestine (%)	3.96 ± 0.15	3.52 ± 0.10	3.61 ± 0.11	0.072
Small intestinal length (cm)	145.78 ± 4.07	142.22 ± 3.40	138.67 ± 3.17	0.400

C diet: 3.0% fish meal and 0% housefly larvae (HL) meal; L diet: 1.5% fish meal and 1.5% HL meal; H diet: 0% fish meal and 3.0% HL meal. Data are presented as mean ± standard error (SE; *n* = 18). Significant differences were set at *p* < 0.05, and trends were defined as 0.05 ≤ *p* < 0.10.

**Table 5:** Selected serum biochemistry parameters.

Parameter	C	L	H	*p*-Value
T-Cho (mg/dL)	82.61 ± 5.25 ^a^	111.61 ± 4.80 ^b^	123.22 ± 4.03 ^b^	<0.001
GOT (IU/L)	112.06 ± 9.44 ^a^	191.44 ± 11.59 ^b^	181.22 ± 15.07 ^b^	<0.001
LDH (IU/L)	494.61 ± 110.14 ^a^	714.88 ± 89.70 ^b^	601.00 ± 112.61 ^a^	0.033
TNF-α (pg/mL)	36.77 ± 3.47 ^a^	37.98 ± 5.14 ^a^	20.70 ± 2.04 ^b^	<0.001

C diet: 3.0% fish meal and 0% housefly larvae (HL) meal; L diet: 1.5% fish meal and 1.5% HL meal; H diet: 0% fish meal and 3.0% HL meal. Data are presented as mean ± standard error (SE; *n* = 18). Different superscript letters indicate significant differences at *p* < 0.05. Significant differences were set at *p* < 0.05, and trends were defined as 0.05 ≤ *p* < 0.10.

**Table 6:** Hematological parameters.

Parameter	C	L	H	*p*-Value
WBC (×10^11^/L)	2.20 ± 0.14	2.20 ± 0.13	2.31 ± 0.06	0.273
RBC (×10^12^/L)	2.50 ± 0.17	2.38 ± 0.13	2.50 ± 0.09	0.063
Lymphocytes (%)	63.35 ± 1.71 ^a^	68.39 ± 0.31 ^b^	68.41 ± 0.50 ^b^	0.014
Granulocytes (%)	27.86 ± 1.74 ^a^	23.20 ± 0.36 ^b^	22.90 ± 0.44 ^b^	0.007
HGB (g/L)	100.33 ± 6.70	100.56 ± 5.32	106.71 ± 3.41	0.277
MCH (pg)	39.98 ± 1.07 ^a^	42.16 ± 0.30 ^b^	42.74 ± 0.42 ^b^	0.012
MCHC (g/L)	311.39 ± 8.94 ^a^	331.25 ± 2.46 ^b^	331.00 ± 3.07 ^b^	0.023

C diet: 3.0% fish meal and 0% housefly larvae (HL) meal; L diet: 1.5% fish meal and 1.5% HL meal; H diet: 0% fish meal and 3.0% HL meal. Data are presented as mean ± standard error (SE; *n* = 18). Different superscript letters indicate significant differences at *p* < 0.05. Significant differences were set at *p* < 0.05, and trends were defined as 0.05 ≤ *p* < 0.10.

**Table 7:** Small intestinal morphometric measurements.

Segment	Parameter	C	L	H	*p*-Value
Duodenum	Villus height (μm)	1606.31 ± 50.01 ^a^	1687.82 ± 41.45 ^ab^	1773.49 ± 53.17 ^b^	0.044
	Crypt depth (μm)	156.16 ± 3.86	148.14 ± 4.07	147.49 ± 4.09	0.175
	V/C ratio	10.39 ± 0.41 ^a^	11.52 ± 0.41 ^ab^	12.18 ± 0.48 ^b^	0.016
Jejunum	Villus height (μm)	1366.19 ± 45.75	1507.93 ± 45.57	1416.72 ± 44.48	0.132
	Crypt depth (μm)	153.71 ± 4.17 ^a^	139.17 ± 4.38 ^b^	131.82 ± 4.24 ^b^	0.004
	V/C ratio	8.92 ± 0.26 ^a^	10.94 ± 0.37 ^b^	10.86 ± 0.37 ^b^	<0.001
Ileum	Villus height (μm)	795.44 ± 24.87 ^a^	883.17 ± 28.53 ^b^	850.73 ± 26.78 ^a^	0.051
	Crypt depth (μm)	144.81 ± 5.09 ^a^	135.91 ± 5.65 ^a^	130.22 ± 3.52 ^b^	0.061
	V/C ratio	5.55 ± 0.19 ^a^	6.64 ± 0.28 ^b^	6.57 ± 0.20 ^b^	0.002

C diet: 3.0% fish meal and 0% housefly larvae (HL) meal; L diet: 1.5% fish meal and 1.5% HL meal; H diet: 0% fish meal and 3.0% HL meal. Data are presented as mean ± standard error (SE; *n* = 18). Different superscript letters indicate significant differences (*p* < 0.05). Significant differences were set at *p* < 0.05, and trends were defined as 0.05 ≤ *p* < 0.10. V/C ratio = villus height to crypt depth ratio.

**Table 8:** Cecal short-chain fatty acid concentrations (μmol/g dry content).

SCFA	C	L	H	*p*-Value
Acetic acid	56.99 ± 9.07	65.25 ± 7.86	49.58 ± 8.11	0.421
Propionic acid	6.50 ± 0.83	6.10 ± 0.66	5.42 ± 0.96	0.095
Isobutyric acid	1.28 ± 0.09 ^a^	1.10 ± 0.10 ^ab^	0.72 ± 0.06 ^b^	0.009
Butyric acid	5.89 ± 1.04	5.61 ± 0.89	6.35 ± 1.11	0.983
Isovaleric acid	1.25 ± 0.10 ^a^	0.98 ± 0.10 ^ab^	0.86 ± 0.16 ^b^	0.002
Valeric acid	0.55 ± 0.38	0.19 ± 0.20	0.00 ± 0.00	0.340

C diet: 3.0% fish meal and 0% housefly larvae (HL) meal; L diet: 1.5% fish meal and 1.5% HL meal; H diet: 0% fish meal and 3.0% HL meal. Data are presented as mean ± standard error (SE; *n* = 18). Different superscript letters indicate significant differences (*p* < 0.05). Significant differences were set at *p* < 0.05, and trends were defined as 0.05 ≤ *p* < 0.10.

**Table 9:** Top 20 significantly different metabolic pathways (ranked by *p*-value).

Pathway ID	Pathway Name	C	L	H	*p*-Value
PWY-6471	Peptidoglycan biosynthesis IV	3409 ± 127 ^a^	4183 ± 145 ^b^	3508 ± 170 ^a^	0.002
GALACTUROCAT-PWY	D-galacturonate degradation I	1726 ± 71 ^a^	2113 ± 92 ^b^	1794 ± 72 ^a^	0.004
PWY-6588	Pyruvate fermentation to acetate VIII	2971 ± 192 ^a^	3810 ± 219 ^b^	3285 ± 196 ^ab^	0.007
PWY-5347	Superpathway of L-methionine biosynthesis	2623 ± 157 ^a^	3366 ± 164 ^b^	2847 ± 149 ^ab^	0.009
P124-PWY	Inosine-5’-phosphate biosynthesis I	2090 ± 148 ^a^	2739 ± 159 ^b^	2269 ± 149 ^ab^	0.010
P125-PWY	Superpathway of purine nucleotide salvage	84 ± 14 ^a^	120 ± 11 ^b^	66 ± 10 ^a^	0.011
PWY-6269	Adenosine nucleotides degradation III	4726 ± 142 ^a^	5378 ± 195 ^b^	4517 ± 194 ^a^	0.013
1CMET2-PWY	N10-formyl-THF biosynthesis	4931 ± 175 ^a^	5663 ± 186 ^b^	4786 ± 227 ^a^	0.013
PWY-5509	Adenosine ribonucleotides de novo biosynthesis	4722 ± 141 ^a^	5371 ± 195 ^b^	4513 ± 194 ^a^	0.013
PWY0-862	(5Z)-dodecenoate biosynthesis I	204 ± 41 ^a^	217 ± 41 ^a^	388 ± 53 ^b^	0.014
MET-SAM-PWY	Superpathway of S-adenosyl-L-methionine biosynthesis	2033 ± 134 ^a^	2669 ± 149 ^b^	2246 ± 123 ^ab^	0.015
DAPLYSINESYN-PWY	L-lysine biosynthesis I	6187 ± 192 ^a^	6983 ± 242 ^b^	5979 ± 277 ^a^	0.015
PYRIDNUCSYN-PWY	NAD biosynthesis I (from aspartate)	4359 ± 153 ^ab^	4775 ± 169 ^a^	3989 ± 193 ^b^	0.016
PWY-5695	Urate biosynthesis/inosine 5’-phosphate degradation	2859 ± 105 ^a^	3252 ± 137 ^b^	2918 ± 135 ^a^	0.016
COMPLETE-ARO-PWY	Superpathway of aromatic amino acid biosynthesis	6884 ± 218 ^a^	7753 ± 266 ^b^	6684 ± 294 ^a^	0.017
LACTOSECAT-PWY	Lactose and galactose degradation I	109 ± 21 ^a^	114 ± 27 ^a^	218 ± 28 ^b^	0.017
PWY-5005	Biotin biosynthesis II	73 ± 15 ^a^	115 ± 14 ^ab^	155 ± 20 ^b^	0.017
P4-PWY	Superpathway of L-lysine, L-threonine and L-methionine biosynthesis I	3070 ± 145 ^a^	3685 ±142 ^b^	3193 ± 151 ^ab^	0.017
PWY-6163	Chorismate biosynthesis from 3-dehydroquinate	6231 ± 201 ^a^	7021 ± 241 ^b^	6039 ± 267 ^a^	0.018
PWY-5505	L-glutamate and L-glutamine biosynthesis	3765 ± 168 ^a^	4487 ± 216 ^b^	3681 ± 184 ^a^	0.018

C diet: 3.0% fish meal and 0% housefly larvae (HL) meal; L diet: 1.5% fish meal and 1.5% HL meal; H diet: 0% fish meal and 3.0% HL meal. Data are presented as mean ± standard error (SE; *n* = 18). Different superscript letters indicate significant differences (*p* < 0.05). Significant differences were set at *p* < 0.05.

**Table 10:** Gross energy, metabolizable energy, and metabolizability of housefly larvae meal.

Parameter	Value
Gross Energy (Mcal/kg)	6.15 ± 0.03
Metabolizable Energy (Mcal/kg)	4.43 ± 0.10
Metabolizability (%)	72.0 ± 1.7

Data are presented as mean ± standard error (SE; *n* = 5).

## Data Availability

The raw data generated and used in this study are available from the corresponding author upon request.

## Supplementary Materials

The following supporting information can be downloaded at: https://www.mdpi.com/article/10.3390/ani16030386/s1, Table S1: Predicted pathway ID raw data.

## Abbreviations

The following abbreviations are used in this manuscript:

FM	Fish meal
GOT	Glutamic oxaloacetic transaminase
HL	Housefly larvae
LDH	Lactate dehydrogenase
ME	Metabolizable energy
SCFA	Short-chain fatty acid
